# Closed‐Loop Recyclable and Extrusion Reprocessable Thermosets Enabled by Guanylthiourea Structure

**DOI:** 10.1002/advs.202410773

**Published:** 2024-11-18

**Authors:** Zhen Yu, Yanlin Liu, Xiangyu Zhou, Yajin Fang, Zhaobin Tang, Jin Zhu, Junping Zhang

**Affiliations:** ^1^ Research Center of Resource Chemistry and Energy Materials, and State Key Laboratory of Solid Lubrication, Lanzhou Institute of Chemical Physics Chinese Academy of Sciences Lanzhou 730000 P. R. China; ^2^ Ningbo Institute of Materials Technology and Engineering Chinese Academy of Sciences Ningbo 315201 P. R. China; ^3^ Center of Materials Science and Optoelectronics Engineering University of Chinese Academy of Sciences Beijing 100049 P. R. China

**Keywords:** composites, closed‐loop recyclable, dynamic covalent polymer, extrusion, guanylthiourea

## Abstract

Plastic recycling is a critical step toward improving waste management and achieving economic recycling. Here, a thermoset crosslinked by guanythiourea structure (GTUH network) is reported, that addresses the recycling issue of thermosets by serial hybridization of thiourea and guanidine urea. The dual dissociative dynamic exchange reaction of guanamine urea and thiourea, combined with non‐covalent hydrogen bonding interactions, endows the network with rapid relaxation ability. GTUH networks, in particular, can be recycled through continuous extrusion processing due to multiple reversible mechanisms, as opposed to hot pressing alone. Even if reprocessed by hot pressing, only 5 min at 140 °C and 10 MPa are required. The oxidation enhancement mechanism of thiourea contributes to maintaining or even improving the mechanical properties of the recycled network. Moreover, the dynamic reactions of guanythiourea structure allow for closed‐loop chemical recycling of the network. Research into recyclable carbon fiber‐reinforced composites indicates promising potential applications for this material in the circular economy and resources.

## Graphical abstract

A novel dynamic polyurea network based on guanythiourea structure is developed, leveraging thiourea and guanidine urea in serial hybridization to achieve rapid relaxation. Various recycling methods are investigated, including compression, welding, extrusion, and chemical recycling. Furthermore, research into recyclable carbon fiber‐reinforced composites indicates promising potential applications for this material in the circular economy and resources.

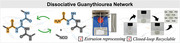

## 1 Introduction

Dynamic covalent polymer networks (DCPNs) utilize reversible chemical reactions that allow them to exhibit fluidity, solid‐state plasticity, and recyclability under various conditions such as temperature, light, or pH.^[^ ^1^ ^]^ The objective of DCPNs is to combine the reprocessing advantages of thermoplastics with the high‐performance characteristics of thermoset materials. DCPNs have been extensively studied across a range of materials including epoxy resins,^[^ ^2^ ^]^ polyurethanes or polyurea (PUA),^[^ ^3^ ^]^ silicone rubber,^[^ ^4^ ^]^ polyolefin rubber,^[^ ^5^ ^]^ and other thermosets or crosslinked elastomers. Integration of dynamic covalent chemistry enables these materials to be reprocessed or recycled.^[^ ^6^ ^]^ Despite their benefits, practical applications of DCPNs often face challenges such as the need for continuous extrusion reprocessing at high temperatures and pressures. Several strategies have been developed to enhance the exchange rates of dynamic bonds, crucial for achieving the low viscosity necessary for efficient continuous reprocessing. These strategies include incorporating catalysts,^[^ ^7^ ^]^ designing networks with low crosslinking density,^[^ ^8^ ^]^ combining multiple dynamic motifs,^[^ ^9^ ^]^ and enhancing the flexibility of chain segments.^[^ ^10^ ^]^ However, challenges persist in maintaining adequate performance metrics such as glass transition temperature and dimensional stability,^[^ ^11^ ^]^ critical for practical applications of DCPNs compared to traditional thermosets. Another consideration is the complexity and increased cost of the synthesis process, necessitating exploration of new methodologies to streamline synthesis processes and reduce costs, making DCPNs economically feasible for diverse applications.^[^ ^12^ ^]^

Guanylthiourea (GTU), with its unique structure combining thiourea and guanidine bonds, is highly reactive in nucleophilic addition reactions, particularly with polyfunctional isocyanates. This reactivity enables the in‐situ formation of dynamic covalent polymer network basing GTU urea structure which can contribute to structural integrity and dynamic properties of the polymer network. In this study, a polymer network, GTUH, based on dual dissociated GTU urea structure, was synthesized by crosslinking GTU with hexamethylene diisocyanate (HDI) via a catalyst‐free, one‐pot method, resulting in a high crosslinking density and outstanding thermodynamic and reprocessing properties. The rapid relaxation of the GTUH network is achieved through successive hybridization with thiourea and guanamine urea, enabling the material to undergo various processing methods such as hot pressing, welding, and even extrusion. The oxidation‐enhanced mechanism of thiourea also ensures the performance of the reprocessed GTUH network. Additionally, the GTUH network can achieve closed‐loop recycling through exchange reactions with GTU. Finally, the potential application of this material in recyclable carbon fiber composites was investigated.

## 2 Results and Discussion

**Figure 1:**
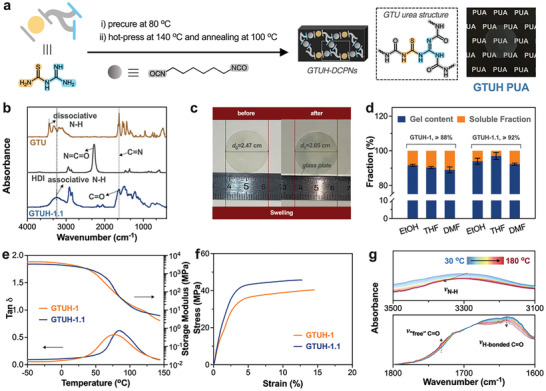
a) Schematic diagram of GTUH networks preparation process and network structure; b) The FTIR spectra of GTU, HDI, and GTUH‐1.1; c) Comparison photos of GTUH‐1.1 before and after immersion in DMF at 50 °C for 48 h; d) Gel content and soluble fraction of GTUH‐1.1 and GTUH‐1 in different solvents; e) DMA and f) stress‐strain curves of GTUH‐1.1 and GTUH‐1; g) The in‐situ FTIR spectra of GTUH‐1.1 in regions 1600 to 1800 cm^−1^ and 3100 to 3500 cm^−1^, with a heating rate of 5 °C min^−1^ from 30 to 180 °C.

GTUH network comprising GTU urea structure was synthesized via nucleophilic addition reaction between GTU and HDI (**Figure** **1**a). The molar ratios of the raw materials were 1:1.1 for GTUH‐1.1 and 1:1 for GTUH‐1 (Table S1, Supporting Information). The TGA curves indicated that after heating the pre‐cured material to 155 °C for 30 min, less than 3% mass loss occurred (Figure S1, Supporting Information), implying effective solvent removal before hot‐press molding. In the FTIR spectra (Figure 1b), characteristic peaks of isocyanates (2200–2400 cm^−1^) and free NH (3440 cm^−1^) disappeared after post‐reaction, while peaks corresponding to C ═ O bonds (1690 cm^−1^) and associative NH groups (3240 cm^−1^) became prominent.^[^ ^13^ ^]^ The peak representing C ═ N bonds (1630 cm^−1^) was retained.^[^ ^13^ ^]^ Swelling experiments in organic solvents confirmed the formation of 3D cross‐linked networks (Figure S2, Supporting Information). For example, the diameter of GTUH‐1.1 increased from 2.47 cm to 2.65 cm in N,N‐dimethylformamide (DMF) at 50 °C for 48 h (Figure 1c), indicating sufficient solvent penetration into the polymer network. Gel content tests in solvents such as ethyl alcohol (EtOH), tetrahydrofuran (THF), and DMF revealed that both GTUH‐1.1 and GTUH‐1 had gel contents exceeding 88% (Figure 1d). Notably, GTUH‐1.1 exhibited over 92% gel content, with less than 8% solubility (Figure 1d), confirming successful formation of highly crosslinking GTUH networks through efficient nucleophilic addition.

The thermal properties of GTUH‐1.1 and GTUH‐1 were evaluated using differential scanning calorimeter (DSC) (Figure S3, Supporting Information) and dynamic mechanical analysis (DMA) (Figure 1e), with summarized data in Table S2 (Supporting Information). GTUH‐1.1 showed a higher *T*_g_ than GTUH‐1, indicating a denser network due to excess HDI ensuring complete reaction during curing. Storage modulus analysis (Figure 1e) showed GTUH‐1 had a decreasing trend at 145 °C, whereas GTUH‐1.1 exhibited stable modulus, suggesting superior degree of crosslinking.^[^ ^8a^ ^]^ Specifically, GTUH‐1.1 at 140 °C demonstrated a crosslinking density of 510 mol m^−3^, significantly higher than GTUH‐1′s 296 mol m^−3^ (Table S2, Supporting Information). The thermal stability of GTUH polyurea was demonstrated characterized with 5% weight loss occurring above 180 °C (Figure S4, Supporting Information). Mechanical properties were evaluated via tensile tests (Figure 1f), revealing GTUH‐1.1 had a Young's modulus of 1822 MPa, elongation at break of 12.7%, and tensile strength of 46 MPa (Table S3, Supporting Information). Strong hydrogen bonding within the GTU urea structure significantly influenced these properties. In‐situ Fourier Transform infrared spectroscopy (FTIR) spectra (Figure 1g) of GTUH‐1.1 indicated temperature‐dependent changes, with NH peaks shifting to higher wavelengths (3100 to 3500 cm^−1^), suggesting H‐bond dissociation. Correspondingly, a decrease in H‐bond C ═ O peak (1650 cm^−1^) and an increase in free C ═ O peak (1730 cm^−1^) was observed. In 2D correlation spectroscopy (Figure S5, Supporting Information), synchronous peaks indicate regions where spectral changes are correlated or synchronized.^[^ ^14^ ^]^ The stronger intensity observed at (1630, 1630 cm^−1^) suggests a higher degree of correlation or simultaneous changes in spectral features associated with H‐bonds in that region. The presence of prominent synchronous peaks implies a robust H‐bonding network within the GTUH material.

**Figure 2:**
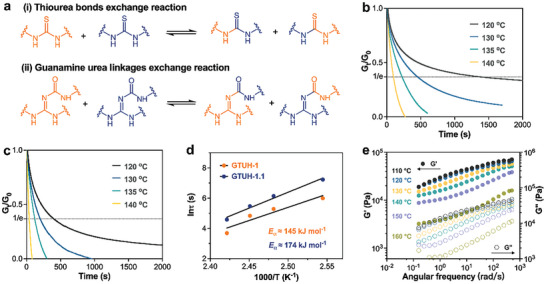
a) Dynamic exchange reaction of thiourea and guanamine urea bonds; Stress relaxation curves of b) GTUH‐1.1 and c) GTUH‐1 at different temperatures; d) Arrhenius plots and the linear fitting of GTUH‐1.1 and GTUH‐1; e) Small amplitude frequency scans profiles for GTUH‐1.1 from 110 to 160 °C.

The GTU urea structure, comprising thiourea and guanidine urea bonds in tandem, combined with rich hydrogen bonding supramolecular interactions, gives highly dynamic characteristics for GTUH network. Two dynamic exchange processes will occur in the network at high temperatures, namely thiourea exchange reaction and guanidine urea exchange reaction, as shown in **Figure** **2**a.^[^ ^3^, ^15^ ^]^ Stress relaxation under a 3% strain at various temperatures follows an exponential decay (Figure 2b,c). The Kohlrausch‐Williams‐Watts (KWW) function was used to fit the relaxation curve and describe the dynamics of network rearrangement (Figure S6, Supporting Information). KWW is a stretched exponential function, $G_{t}/\;\;\;G_{0}=e^{-{\left(t/\tau \right)}^{\beta }}$, where t is the relaxation time, G_t_ is the modulus of the sample after relaxation time, G_0_ is the initial modulus of the sample. The characteristic relaxation time *τ* signifies the duration for stress to decrease to 1/e of its original value after force cessation. The exponent *β* (where 0 < *β* ≤ 1) controls the shape of the stretched exponential decay, reflecting the width of the relaxation distribution. As the temperature increases, the value of *β* becomes larger, indicating a narrowing of the relaxation time distribution (Table S4, Supporting Information). When *β* ═ 1, it signifies a single relaxation time, and the equation can be simplified to the classical Maxwell model.^[^ ^16^ ^]^ Increasing the temperature not only narrows the distribution range of relaxation times but also accelerates the relaxation process. For instance, in the transition from 120 to 140 °C, GTUH‐1.1 exhibits a decrease in relaxation time from 1380 s to 98 s, while GTUH‐1 reduces from 400 to 40 s (Figure 2b,c). The crosslinking density of the network affects relaxation speed, generally correlating higher densities with longer relaxation times. Calculated via the Arrhenius formula (Figure 2d), exchange activation energies indicate that denser networks require more energy for exchange reactions (174 kJ mol^−1^ for GTUH‐1.1, 145 kJ mol^−1^ for GTUH‐1). As depicted in Figure 2e, the storage modulus (G') of GTUH‐1.1 diminishes with rising temperature, decreasing from 19 104.5 Pa at 110 °C to 3226.68 Pa at 160 °C by rotary rheometer. The variation curves of the storage modulus as a function of temperature by DMA can also reflect the dissociation behavior of the GTUH network. In Figure S7 (Supporting Information), taking GTUH‐1 as typical, it is evident that modulus undergoes two sharp drops, the first corresponding to the glass transition behavior and the second to the topological transition behavior of the dissociative network. The GTUH network exhibits sensitivity to temperature fluctuations due to the dual dissociative exchange mechanism of the GTU urea structure. The contribution of guanamine urea and thiourea bonds to the network topology transformation was further studied and discussed. We synthesized polymer networks GxTy with different bond ratios according to previously reported method (Figure S8 and Table S4, Supporting Information), where *x* and *y* represent the ratios of guanamine urea and thiourea in networks, specifically 10:0, 7:3, 5:5, 3:7, and 0:10. For example, at 140 °C, the *τ* of G7T3 was the shortest of only 67 s (Figure S9, Supporting Information), and its exchange activation energy (*E*_a_) was the lowest of 81 kJ mol^−1^ (Figure S10, Supporting Information). The *τ*s for G10T0 and G0T10 were 124 s and 6273 s, and *E*_a_s for them were 96 kJ mol^−1^ and 149 kJ mol^−1^. An appropriate bond ratio can synergistically promote the transformation of network topology. Considering the structural characteristic of GTU sharing nitrogen, the dissociation behavior within GTUH network is more likely to occur and may even lead to collapse with increasing temperature. Increased dissociation at temperatures beyond specific thresholds (approximately 160 °C for GTUH‐1 and 180 °C for GTUH‐1.1) leads to network collapse (Figure S11, Supporting Information), though Arrhenius behavior persists below these temperatures (Figure 2d). The GTUH network leverages its innovative guanamine urea and thiourea dual dissociation mechanisms, coupled with structural designs that incorporate shared nitrogen atoms, to exhibit an extraordinary level of dynamic tunability. Crucially, high degree of dynamism proves to be indispensable for the continuous processing of dynamic polymer networks, significantly outperforming other single dynamic covalent systems (Table S6, Supporting Information). The serial arrangement of thiourea and guanidine urea bonds within the GTUH network facilitates efficient reprocessing and continuous material processing.

**Figure 3:**
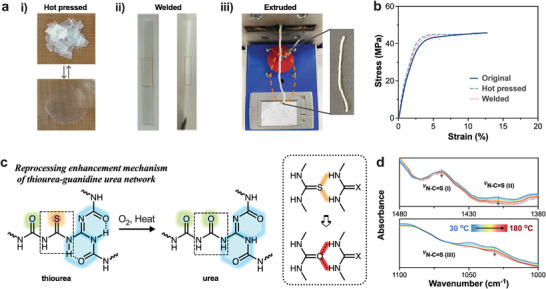
a) Digital photos of (i) compression, (ii) welded and (iii) extrusion reprocessing of GTUH‐1.1; b) Representative stress‐strain curves of the original and recycled GTUH‐1.1; c) reprocessing enhanced mechanism of GTUH network; d) The in‐situ FTIR spectra of GTUH‐1.1 in the region of 1000 to 1100 cm^−1^ and 1380 to 1480 cm^−1^.

The rapid reversible reaction of dynamic covalent bonds in the network can endow thermosets with reprocessing properties, and hydrogen bonding supramolecular interactions can accelerate this process. The majority of reported dynamic covalent polymer networks are reprocessed by hot pressing, with only a few instances of reprocessing by extrusion due to the networks' limited dynamism. Various processing methods were employed for the reprocessing of GTUH polyurea, including compression molding, welding, and extrusion (**Figure** **3**a). In compression molding, GTUH‐1.1 film segments are cut and hot‐pressed to form complete films. Effective reprocessing occurs at 140 °C and 10 MPa for 5 min, finally obtaining films of high uniformity and transparency (Figure 3a). Mechanical properties such as tensile strength and Young's modulus remained robust or even improved post‐compression due to thiourea oxidation to urea, enhancing hydrogen bonding within the network (Figure 3b; Table S3, Supporting Information).^[^ ^11^ ^]^ Observation in Figure 3d shows characteristic peaks of thiourea bonds at specific wavenumbers (1500–1430 cm^−1^, 1450–1350 cm^−1^, and 1120–1050 cm^−1^) decreasing in intensity, indicating a reduction in thiourea bonds with increasing temperature within the GTUH network.^[^ ^17^ ^]^ As the temperature decreases, the characteristic peaks of N‐C ═ S (I) (1500–1430 cm^−1^) and N‐C ═ S (III) (1120–1050 cm^−1^) show no significant changes, while N─C ═ S (II) (1450–1350 cm^−1^) exhibits a slight increase (Figure S13, Supporting Information). Additionally, for the free C ═ O (1730 cm^−1^) peak, which theoretically should decrease during cooling in the absence of other influences, there is essentially no change, and it even shows a trend of increasing (Figure S13, Supporting Information). This observation provides further support for the oxidative behavior of thiourea in the GTUH network during the heating process. The observed color change of the sample in the swelling experiment provides further evidence. After 48 h of immersion in oxidizing solvents such as THF, the sample began to turn yellow, suggesting that the GTUH network's thiourea linkages had been oxidized to urea bonds (Figure S2, Supporting Information). In contrast, no color change was detected in the sample immersed in non‐oxidizing solvents such as DMF (Figure S2, Supporting Information). In comparison to thiourea bonds, urea bonds generate stronger hydrogen bonds, which improves the mechanical properties of the samples after cyclic processing (Figure 3c). Welding GTUH polyurea involves heating to activate network exchange sites, enabling the welding of different sheets (Figure 3a). Post‐welding, GTUH‐1.1 exhibits strength comparable to the original sample, highlighting good viscoelastic properties (Figure 3b). Unlike polymer networks that only contain guanamine urea type or thiourea type interactions, the GTU network, which has the sharing nitrogen structural features and benefits from the synergy of guanamine urea and thiourea bonds, exhibits easier and more dissociation behavior. In Figure S14 (Supporting Information), it is observed that the complex viscosity of G10T0 and G0T10 remains above 1000 Pa s throughout the temperature increase to 180 °C. However, as the temperature rises from 140 to 160 °C, the complex viscosity of GTUH‐1.1 drops from above 1000 Pa s to below 100 Pa s. This allows for the continuous processing of the GTUH‐1.1 network within the temperature range of 140 °C to 160 °C. Finally, micro‐extrusion at 160 °C with a 2.16 kg load was explored for reprocessing GTUH‐1.1 (Figure 3a). Despite the presence of defects or cracks on the extrudate surface that severely compromise mechanical properties, thermal stability is somewhat preserved (Figure S15, Supporting Information). The experiment demonstrates promising potential for reprocessing dynamic GTUH networks through extrusion.

**Figure 4:**
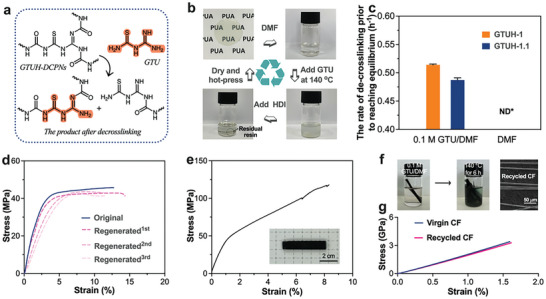
a) De‐crosslinking of GTUH networks; b) The de‐crosslinking for GTUH‐1.1 at 140 °C in 0.1 M GTU/DMF, with the corresponding amount of HDI added for re‐crosslinking; c) The degradation rate prior to reaching equilibrium of GTUH polyurea; d) Representative stress‐strain curves of original and regenerated resins; e) Stress‐strain curve and photo of CF composites prepared with GTUH‐1.1 as resin matrix; f) Photos of CF composites degraded at 0.1 M GTU/DMF at 140 ° C for 6 h, and SEM image of recycled CF; g) Stress‐strain curve of virgin and recycled CF.

The effective dissociation and reconstruction of dynamic covalent bonds may achieve closed‐loop recovery of thermosets.^[^ ^18^ ^]^ In our preliminary work, we presented a network with novel guanidine urea dynamic bonds, which was found to be unable to achieve closed‐loop recovery through the dissociation of dynamic bonds. The reason for this was analyzed to be the insufficient density of dynamic covalent sites in the network. GTUH materials possess a distinctive double dissociation characteristic that facilitates their chemical recovery through de‐crosslinking networks using small molecules. Specifically, thiourea and guanamine urea bonds in GTU urea structure enable network fragmentation and reorganization via exchange reactions (**Figure** **4**a). De‐crosslinking experiments demonstrated that GTUH can be effectively degraded by heating ≈10 mg of the material in a 0.1 M GTU/DMF solution at 140 °C for 2 h (Figure 4b). A control experiment depicted in Figure S16 (Supporting Information) subjected the sample to 140 °C in DMF for 2 h without observable change. GTUH‐1 exhibited a faster decrosslinking rate under similar conditions (Figure 4c). To regenerate GTUH polyurea, the degradation solution can be re‐crosslinked by adding the appropriate amount of HDI (Figure 4a). For example, adding 78 mg of HDI to the degradation solution of GTUH‐1.1 successfully regenerated polyurea after a curing process. The mechanical properties and chemical structure of the regenerated material were well preserved (Figure 4d; Figure S17, Supporting Information). Furthermore, GTUH polyurea was utilized as a resin matrix in carbon fiber (CF) composites, showcasing impressive mechanical properties with a tensile strength of 110 ± 11 MPa, Young's modulus of 4.8 ± 0.2 GPa, and elongation at break of 8.1 ± 0.4% (Figure 4e). CF composite samples were welded at a temperature of 140 °C, covering a contact area of ≈100 mm^2^. The samples experienced failure at the shear displacement of 0.0023 ± 0.0007 mm mm^−1^, demonstrating the lap shear strength of 0.96 ± 0.04 MPa (Figure S18, Supporting Information). The same decrosslinking method employed for material recovery was also used to remove the resin matrix from CF surfaces (Figure 4f). This process successfully left no residue on the CF surface, as observed by scanning electron microscope (SEM) (Figure 4f), while maintaining the chemical structure and mechanical properties of the recovered carbon fiber (Figure 4g; Figures S19‐S21, Supporting Information). In the Raman spectra of both virgin and recycled carbon fibers (Figure S19a, Supporting Information), coincident peaks can be observed at 1350 cm^−1^, 1580 cm^−1^, and 2700 cm^−1^. In the 1D wide‐angle X‐ray scattering (1D WDX) files (Figure S19b, Supporting Information) of both virgin and recycled carbon fibers, peaks typically corresponding to the (002) and (100) crystal planes appear at around 2θ ═ 22° and 44°. Additionally, in the 2D WDX patterns (Figures S20 and S21, Supporting Information), the spatial distribution of diffraction patterns before and after recycling remains largely consistent. Overall, the capability of GTUH materials to undergo controlled degradation and regeneration underscores their potential for sustainable applications in advanced materials science.^[^ ^19^ ^]^

## 3 Conclusion

The GTUH network demonstrates exceptional performance and rapid reprocessing capabilities, leveraging to its unique dual‐dissociation GTU urea structure. The structure facilitates rapid relaxation within the GTUH network, promoting effective reprocessing and recycling, which is attributed to successive hybridization of thiourea and guanidine bonds from the GTU urea structure. The focus is on the continuous extrusion and closed‐loop chemical recycling of the GTUH network. Notably, due to the advanced thiourea oxidation mechanism, this can restore the mechanical properties of the GTUH network to, or even beyond, their original state. Additionally, research on recyclable carbon fiber reinforced composites indicates that this multifunctional material aligns well with the current context of a circular economy.

## Conflict of Interest

The authors declare no conflict of interest.

## Supporting information



Supporting Information

## Data Availability

The data that support the findings of this study are available from the corresponding author upon reasonable request.
