# Supplementary material for: Closed‐Loop Recyclable and Extrusion Reprocessable Thermosets Enabled by Guanylthiourea Structure
Source: Adv Sci (Weinh). 2024 Nov 18;11(48):2410773. doi: 10.1002/advs.202410773 (PMC11672245; doi:10.1002/advs.202410773)
Supplement: Supplementary file 1 — Supporting Information [file ADVS-11-2410773-s001.docx]

**Supporting information**

**Closed-loop Recyclable and Extrusion Reprocessable Thermosets Enabled by Guanylthiourea Structure**

Zhen Yu ^a,c^, Yanlin Liu^b*^, Xiangyu Zhou^b,c^, Yajin Fang^b^, Zhaobin Tang^b*^, Jin Zhu^b^, and Junping Zhang^a,c^^*^

^a^Research Center of Resource Chemistry and Energy Materials, and State Key Laboratory of Solid Lubrication, Lanzhou Institute of Chemical Physics, Chinese Academy of Sciences, Lanzhou 730000, P.R. China;

^b^Ningbo Institute of Materials Technology and Engineering, Chinese Academy of Sciences, Ningbo 315201, P.R. China;

^c^Center of Materials Science and Optoelectronics Engineering, University of Chinese Academy of Sciences, Beijing 100049, P.R. China;

*Corresponding authors: (Yanlin Liu) E-mail: [liuyanlin@nimte.ac.cn](mailto:liuyanlin@nimte.ac.cn); (Zhaobin Tang) E-mail: [tangzhb04@nimte.ac.cn](mailto:tangzhb04@nimte.ac.cn); (Junping Zhang) E-mail: jpzhang@licp.cas.cn.

**Supplementary Experimental Sections**

**Materials.** Guanylthiourea (GTU), thiourea and dimethyl sulfoxide (DMSO) were purchased from Aladdin Reagent Co., China. Hexamethylene diisocyanate (HDI), hexamethylene diisocyanate trimer (tri-HDI) and 1-acetylguanidine were bought from Macklin Co., China. Methanol, ethanol (EtOH), acetone, methyl ethyl ketone (MEK), tetrahydrofuran (THF) and *N*,*N*-Dimethylformamide (DMF) were all from Sinopharm Chemical Reagent Co., Ltd., China. The T300-3K carbon fiber cloth was obtained from TORAY Co., Ltd., Japan. All raw materials were used as received.

**Preparation of GTUH Polyurea and CFRP.** GTU and HDI were mixed in DMF at 80 ℃ for 30 min with protection in nitrogen atmosphere. After the pre-reaction, most of the DMF solvent was removed using a rotary evaporator at 80 ℃. The mixture was then placed in a vacuum oven and heated at 80 ℃. Once the mixture has gelled, it was subjected to hot-pressing which conducted at 140 ℃, 10 MPa. The hot-pressed material was then post-cured in a vacuum oven at 100 ℃ for 6 h. The post-curing ensures that any remaining reactive groups are fully reacted. After the post-curing step, the final products, referred to as GTUH, were obtained. All GTUH polyurea were fed according to **Table S1**. About 10g of precured GTUH-1.1 and 5 pieces of T300-3K carbon fiber cloth were layered in the same direction in a mold sized 100 mm x 100 mm. The layers were compacted together. It ensures that the resin is evenly distributed and adheres well to the carbon fiber cloth. The sample was sealed and then treated at 80 °C for 4 h. The sample is then molded at 140 °C under a pressure of 20 MPa. The molded sample was cured in a vacuum oven at 100 °C for 6 h and finally obtained a CFRP of about 2.7 mm thickness.

**Swelling experiment.** About 100 mg cured samples were soaked in methanol, EtOH, acetone, MEK, THF and DMF at 50 °C for 48 h. Wipe the solvent on the surface of the sample. The swelling ratio is calculated as $\frac{m_{1}}{m_{0}}\times100\%$. *m*_0_ is the initial mass, and *m*_1_ is the final mass after wiping.

**Gel content test.** About 100 mg samples were soaked in EtOH, THF and DMF at 50 °C for 72 h. Then dry in a vacuum oven at 80 °C for 24 h. The gel content is calculated as $\frac{m_{1}}{m_{0}}\times100\%$. *m*_0_ is the initial mass, and *m*_1_ is the final mass after drying.

**Chemical recycling of GTUH network.** About 10 mg samples were de-crosslinked in 6 mL 0.1 M GTU/DMF solution by heating of the material at 140 ℃ for 2 h. The de-crosslinking rate is calculated as $\frac{m_{0}-m_{1}}{m_{0}\times t}$. *m*_0_ is the initial mass, *m*_1_ is the final mass after degradation and drying, and *t* stands for degradation time.

**Characterization.** The FTIR spectra of powder and liquid samples were recorded by the NICOLET 6700 (THermo, USA) fourier transform infrared spectrometer. The FTIR spectra of the film sample was recorded by the Cary660+620 (Agilent, USA) microscopic infrared spectrometer spectrometer. The *in-situ* FTIR spectra of the cured were heated from 30 °C to 180 °C and down to 30 °C by KBr tablet preparation at a rate of 5 °C min^-1^ and recorded once per minute. The DSC curves were written by the DSC3+ (Mettler, Switzerland) differential scanning calorimeter. From -40 °C to 140 °C at a rate of 20 °C min^-1^, followed by a 5 min hold at 140 °C. From 140 °C back to -40 °C at a faster rate of 50 °C min^-1^. From -40 °C to 200 °C at a rate of 20 °C min^-1^. The glass transition temperature (*T*_g_) of the sample is identified as the peak temperature of the differential curve during the second heating cycle. The TGA curves were written by TGA/DSC1 (METTLER TOLEDO, China) from 50 °C to 800 °C with 20 °C min^-1^ in a N_2_ atmosphere. Tensile tests were recorded with the Z1.0 (Zwick, Germany) universal testing machine. The DMA curves were written by DMAQ850 (TA, USA) dynamic mechanical analyzer from -50 °C to 150 °C with 5 °C min^-1^ with an amplitude of 10 *u*m, a frequency of 1 Hz, and a preload of 5 × 10^-3^ N. The crosslinking density (*v*_e_) was estimated by **equation (S1)**.^[20]^

$$v_{e}=\frac{E^{'}}{3RT} (S1)$$

where *E*' is the corresponding storage modulus at 140 °C, and *R* is the gas constant. Stress relaxation experiments were tested by DMAQ850 at 120 °C, 130 °C, 135 °C, and 140 °C with a constant strain of 3% and a preload of 5 × 10^-3^ N. The relaxation time (*τ*) of all samples are utilized to estimate the activation energy (*E*_a_) of the network exchange reaction by **equation (S2).**^[21]^

$\tau^{*}(T)=\tau_{0}e^{\frac{E_{a}}{RT}}$ (S2)

where *τ*_₀_ represents the pre-factor, *R* is the gas constant, and *T* is the experimental temperature. The rheological behavior of the cured was tested by the HR-3 (TA, USA) rotary rheometer. Small amplitude temperature ramp profiles were recorded from 30 to 200 ℃ at 5 ℃ min^-1^ with an axial force fixed at 2 N in a N_2_ atmosphere. Small amplitude frequency scanning profiles were recorded from 0.1 to 500 rad with an axial force of 2 N at 110 ℃, 120 ℃, 130 ℃, 140 ℃, 150 ℃ and 160 ℃ in a N_2_ atmosphere. The tensile tests of the cured were carried out on the Z1.0 (Zwick, Germany) universal testing machine. The stress-strain and force-shear displacement curves of the composite were recorded by Zwick/Roell Z030 (Zwick, Germany). The tensile tests of CFs were carried out on the FAVIMAT+ (TEXTECHNO, German). The surface morphology of CFs was observed by MAGNA (TESCAN, Czech Republic) scanning electron microscopy. Raman spectra were recorded by the Renishaw inVia Reflex (Renishaw, UK) confocal micro-Raman spectrometer. WDX spectra of CFs were recorded by Xeuss 3.0 UHR (XENOCS SAS, France) X-ray scatterer.

**Statistical Analysis.** Statistical analysis was performed using GraphPad Prism 10 (GraphPad Software Inc., San Diego, USA). Simple linear regression was used to model and calculate the activation energy of the network exchange reactions. The row statistics method was applied to compute the gel content, the rate de-crosslinking prior to reaching equilibrium, and mechanical properties of the CF composites, with the results presented as mean ± SD, where n=2.

**Supplementary Figures**

**
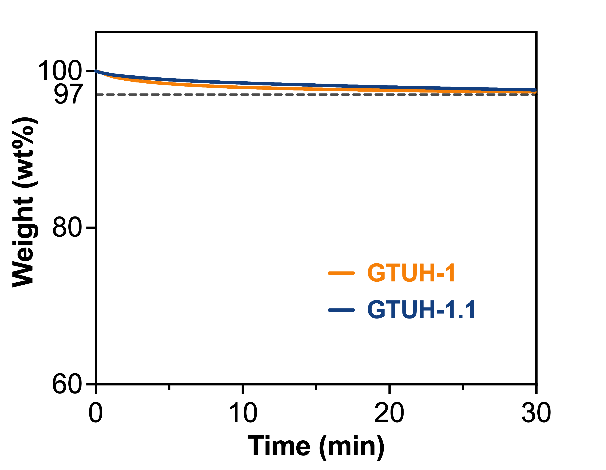
**

**Figure S1.** TGA curves of pre-cured GTUH-1.1 and GTUH-1 at 155 °C for 30 min.

**
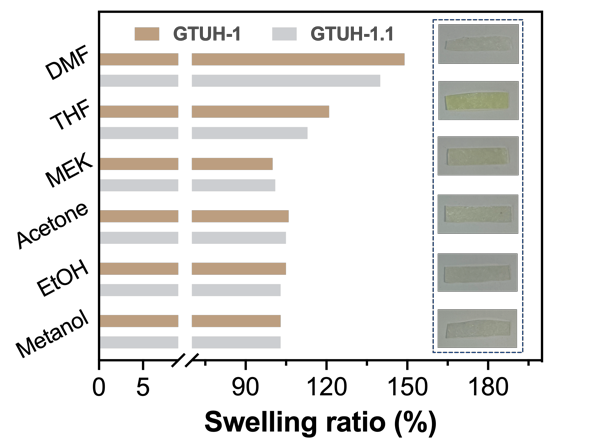
**

**Figure S2****.** Swelling ratio of GTUH-1.1 and GTUH-1(Insert: Photo of GTUH-1.1 after swelling).


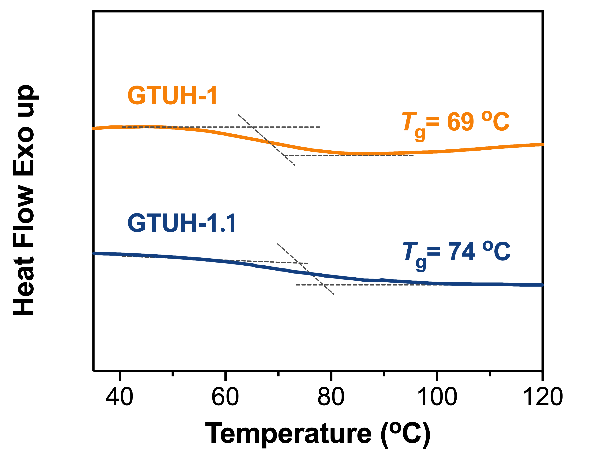


**Figure S3****.** DSC curves of GTUH-1.1 and GTUH-1.

**
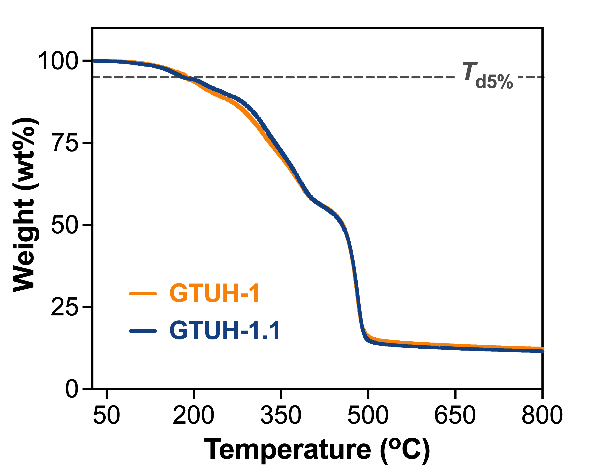
**

**Figure S4.** TGA curves of GTUH polyurea.


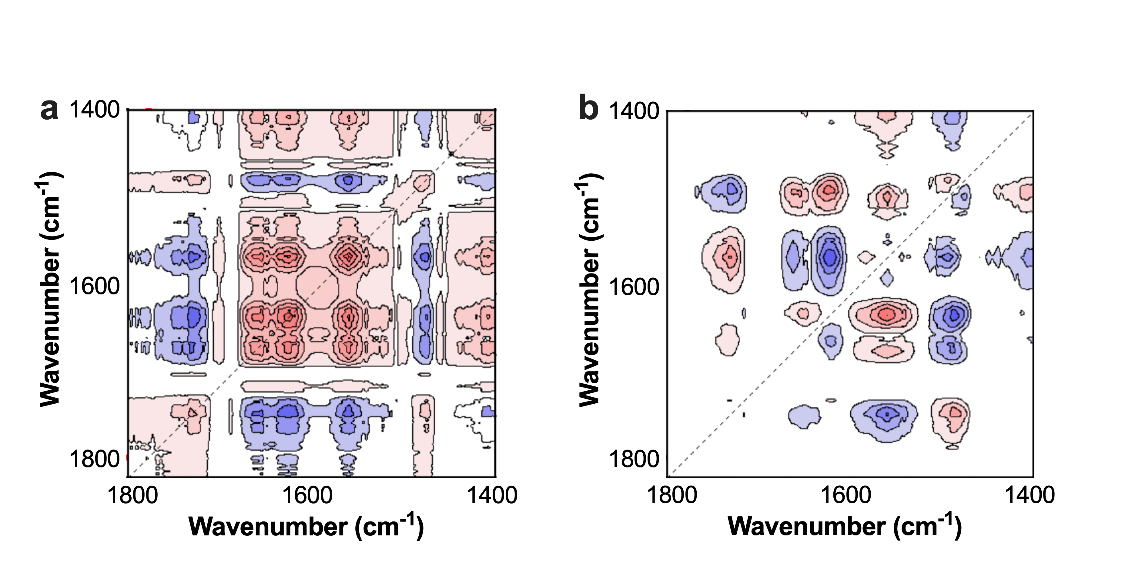


**Figure** **S5.** Synchronous and asynchronous 2D correlation spectra in regions from 1400 cm^-1^ to 1800 cm^-1^, in which red and blue represent positive and negative intensities, indicating regions where spectral changes are correlated or anti-correlated.


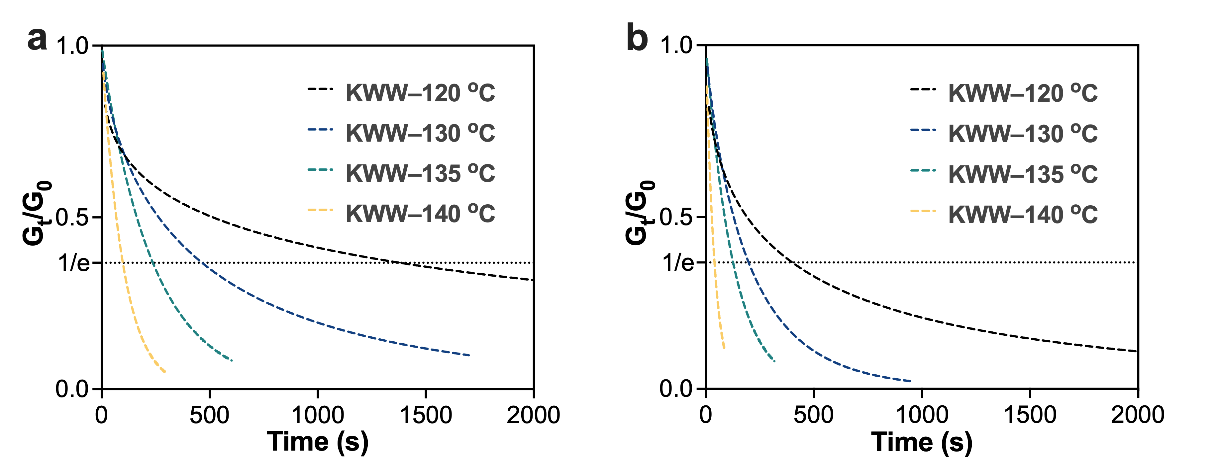


**Figure S6.** The stress relaxation curves of GTUH-1.1 and GTUH-1 networks at different temperatures were fitted with KWW function.


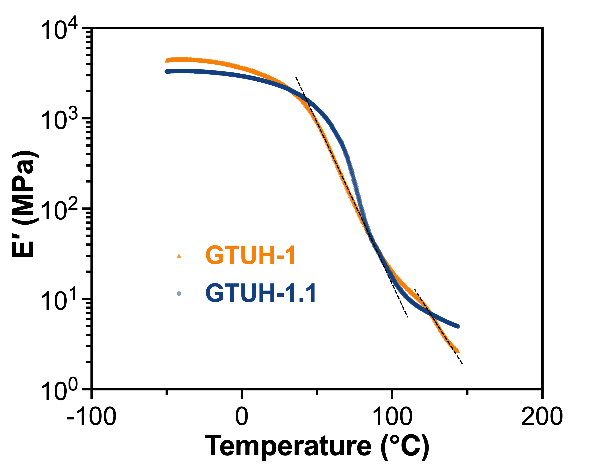


**Figure S7.** The storage modulus (E') as a function of temperature varies from -50 to 150 ℃ by the DMA method.


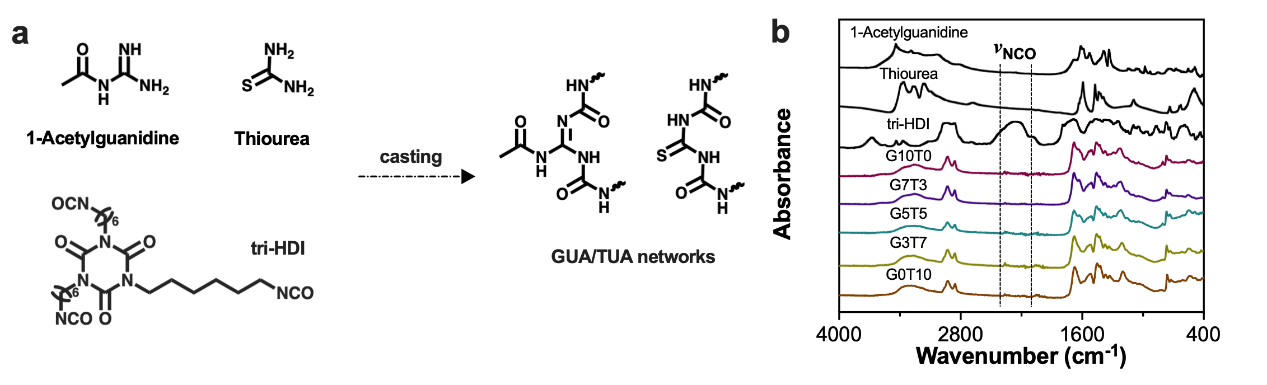


**Figure S8.** (a) GxTy polymers preparation process and network structure (x and y are the molar ratios of 1-acetylguanidine to thiourea, which are 10:0, 7:3, 5:5, 3:7, and 0:10); (b) FTIR spectra of polymers and raw materials. It should be noted that for polymer networks involving thiourea structures, DMF should be replaced with DMSO. Other than this alteration, the synthesis method remains consistent with the reported^[3a]^.

**
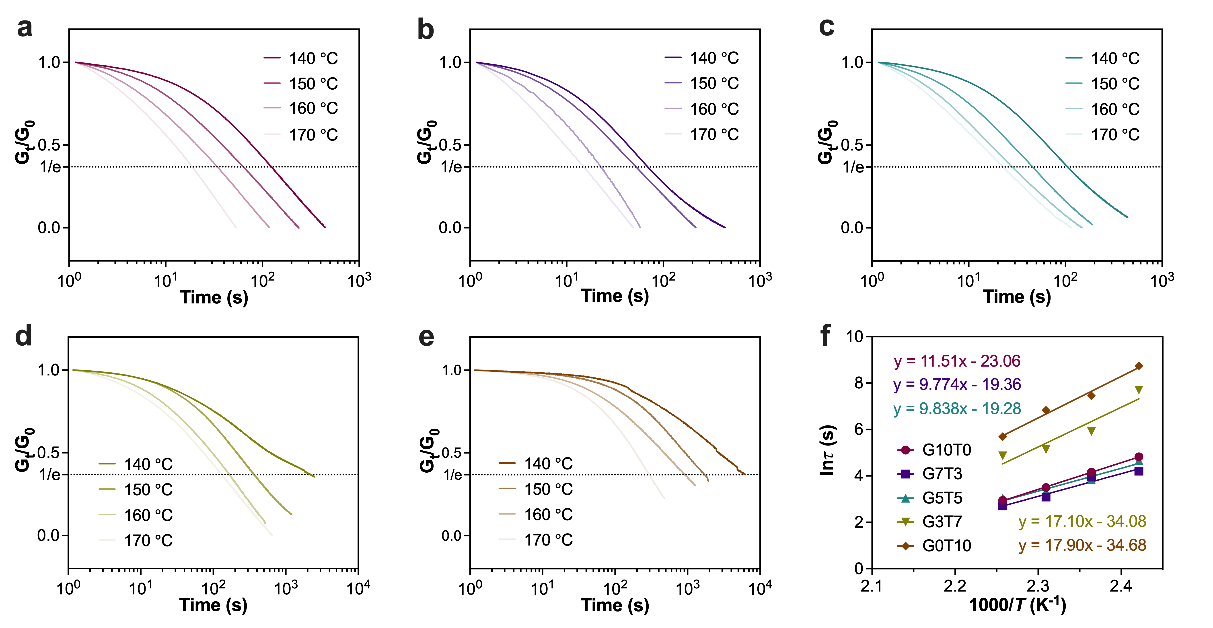
**

**Figure S9.** Stress relaxation curves of (a) G10T0, (b) G7T3, (c) G5T5, (d) G3T7 and (e) G0T10 at 140 °C, 150 °C, 160 °C and 170 °C; (f) Arrhenius plots and the linear fitting of GxTy.


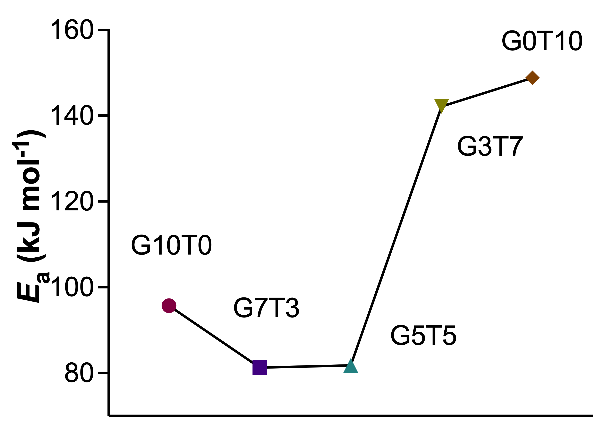


**Figure S10.** Exchange reaction activation energy of GxTy polymer networks.


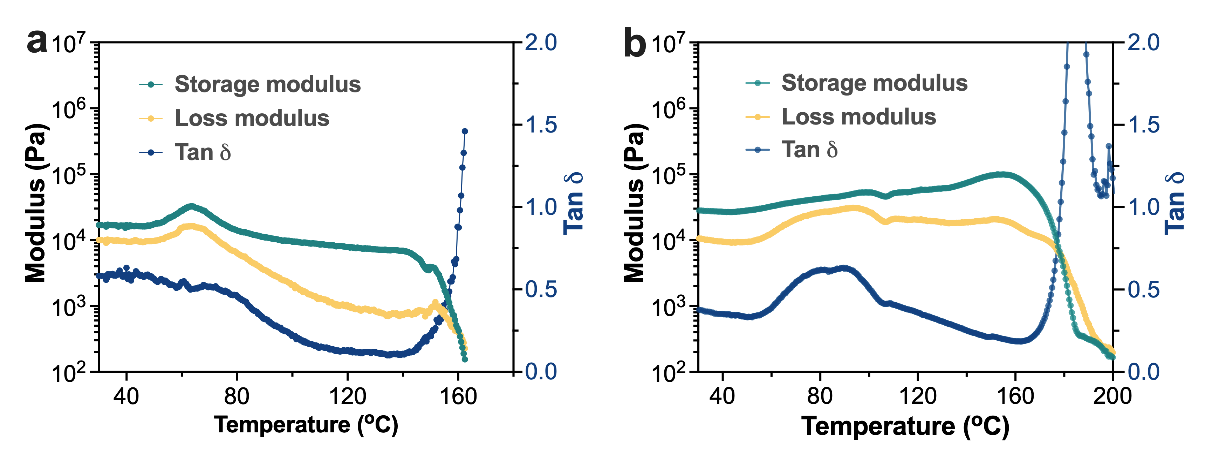


**Figure S11.** Small amplitude temperature scanning profiles of (a) GTUH-1 and (b) GTUH-1.1 polyurea.

**
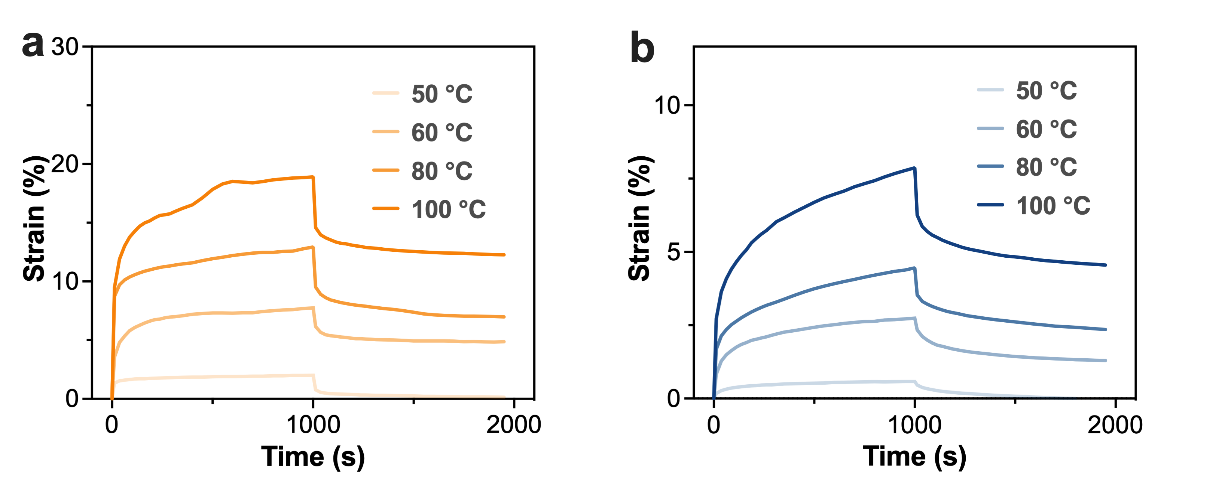
**

**Figure S12.** Creep and creep recovery curves of GTUH polyurea.


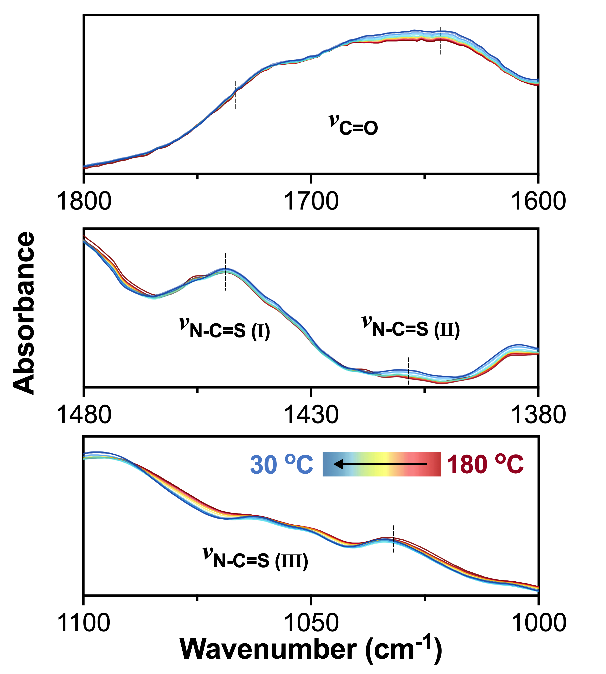


**Figure S13.** The *in-situ* FTIR spectra of GTUH-1.1 in the region of 1000 to 1100 cm^-1^ and 1380 to 1480 cm^-1^, and 1600 to 1800 cm^-1^ from 180 °C to 30 °C.

**
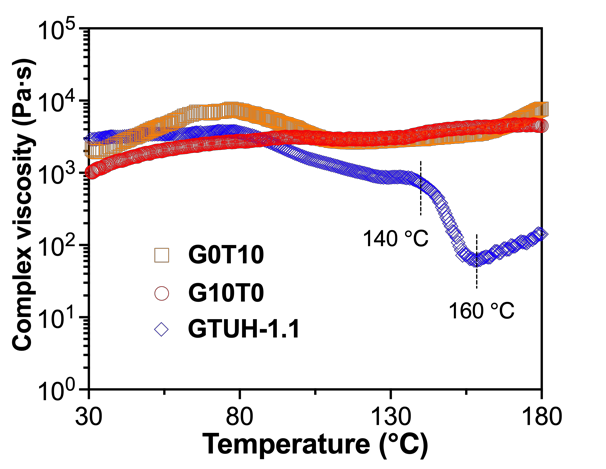
**

**Figure S14.** The variation curves of complex viscosity with temperature for G10T0, G0T0, and GTUH-1.1 were obtained through rotary rheometer.


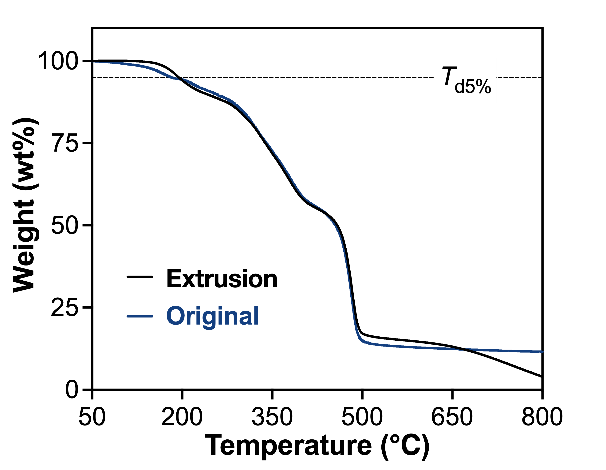


**Figure S15.** TGA curves of extruded and original GTUH-1.1.


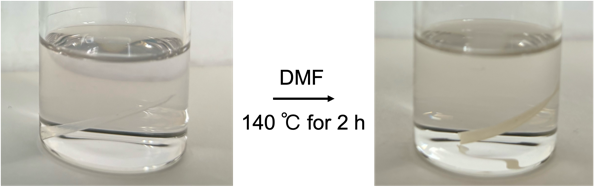


**Figure S16.** Photos of GTUH before and after soaking in DMF at 140 °C for 2 h.

**
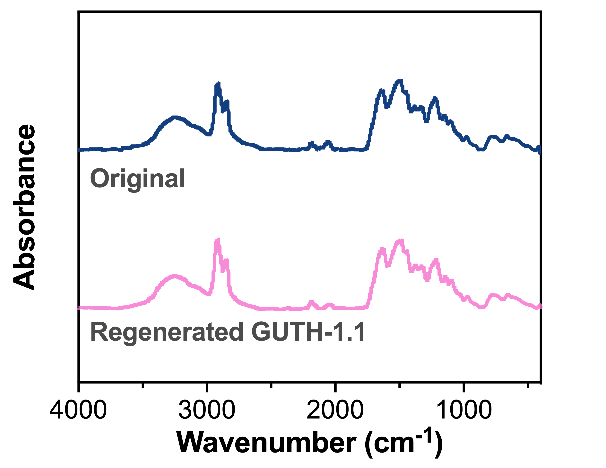
**

**Figure S17.** The FTIR spectra of original and regenerated resins.


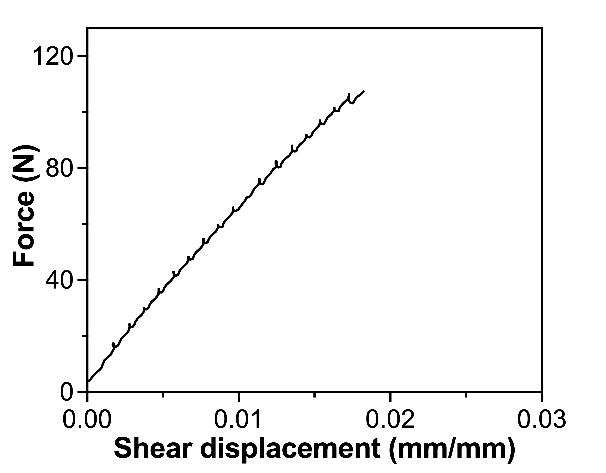


**Figure S18.** Force-shear displacement curve of CF composites.


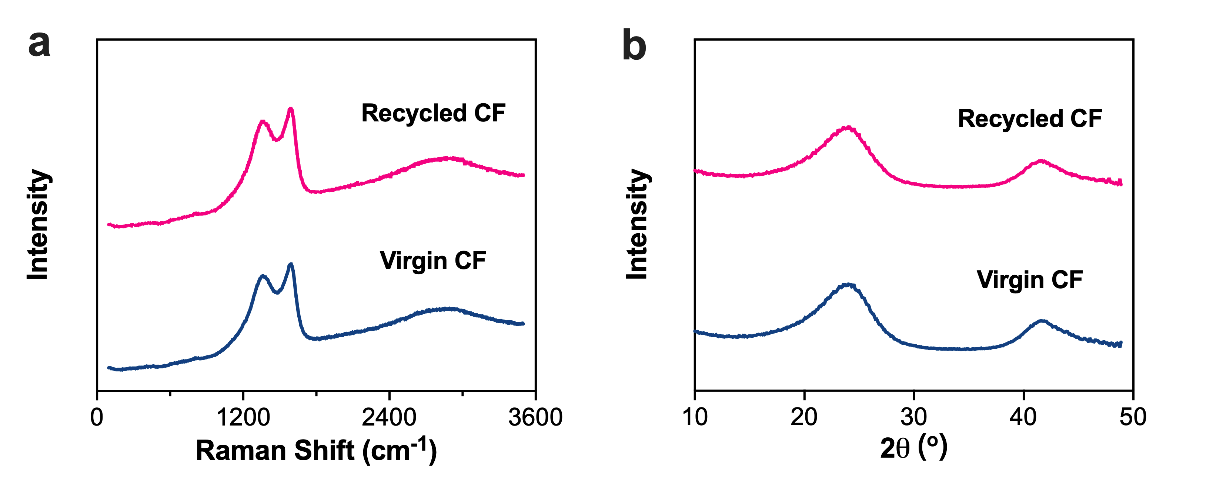


**Figure S19.** The WDX and Raman spectra of virgin and recycled CF.


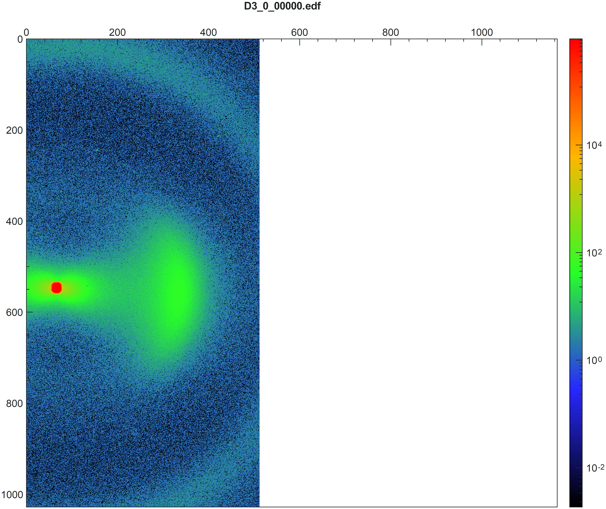


**Figure S20.** The 2D WDX profile of recycled CF.


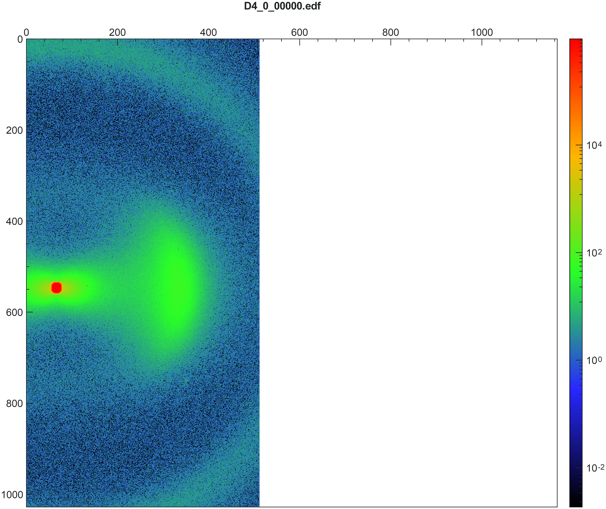


**Figure S21.** The 2D WDX profile of virgin CF.

**Supplementary Tables**

**Table S1**. Composition of different GTUH polyurea.

| Sample | Composition (g) | |
| --- | --- | --- |
|  | GTU | HDI |
| GTUH-1 | 2.36 | 5.04 |
| GTUH-1.1 | 2.36 | 5.54 |

**Table S2**. Glass transition temperature (*T*_g_), storage modulus at *T =* 140 °C (*E*’) and cross-link density (*v*_e_) of GTUH polyurea.

| Sample | *T*_g_ (°C) | | *E*’ (MPa) | *v*_e_ (mol m^-3^) |
| --- | --- | --- | --- | --- |
|  | DSC | DMA |  |  |
| GTUH-1 | 69 | 78 | 3.05 | 510 |
| GTUH-1.1 | 74 | 85 | 5.25 | 296 |

**Table S3**. Young's modulus, tensile strength, and elongation at break of GTUH polyurea.

| Sample | Young's modulus (MPa) | tensile strength (MPa) | elongation at break (%) |
| --- | --- | --- | --- |
| GTUH-1 | 1416 | 40 | 14.5 |
| GTUH-1.1 | 1822 | 46 | 12.7 |
| Hot pressed GTUH-1.1 | 1886 | 46 | 11.7 |
| Welded GTUH-1.1 | 1865 | 44 | 5.9 |
| Regenerated GTUH-1.1 | 1666 | 41 | 14.3 |

**Table S4**. Stress relaxation times determined from the 1/e method and from KWW fits.

| GTUH-1 | 120 °C | 130 °C | 135 °C | 140 °C |
| --- | --- | --- | --- | --- |
| *τ*/s | 400 | 198 | 126 | 40 |
| *τ*_KWW_/s | 401 | 198 | 126 | 40 |
| *β* | 0.49 | 0.85 | 1.00 | 1.00 |
| GTUH-1.1 | 120 °C | 130 °C | 135 °C | 140 °C |
| *τ*/s | 1381 | 466 | 238 | 98 |
| *τ*_KWW_/s | 1377 | 465 | 237 | 98 |
| *β* | 0.37 | 0.65 | 0.98 | 1.00 |

**Table S5**. Composition of GxTy networks.

| Sample | Composition (g) | | |
| --- | --- | --- | --- |
|  | 1-acetylguanidine | Thiourea | tri-HDI |
| G10T0 | 0.303 | 0 | 1.008 |
| G7T3 | 0.212 | 0.069 |  |
| G5T5 | 0.152 | 0.114 |  |
| G3T7 | 0.091 | 0.160 |  |
| G0T10 | 0 | 0.228 |  |

**Table S6**. Comparison of double dissociated GTUH networks with other mono dynamic covalent systems.

| Network type | Samples | Applied strain | τ at T_max_ | E_a_ (kJ mol^-1^) | Hot-pressing | Extrusion | |
| --- | --- | --- | --- | --- | --- | --- | --- |
| guanamine urea/thiourea | GTUH-1.1 | 3% | 98 s at 140 °C | 174 | 140 °C, 10 MPa for 5 min | 160 °C with a 2.16 kg load |  |
| guanamine urea | G1T0 | 3% | 19 s at 170 °C | 96 | —— | —— |  |
| thiourea | G0T1 | 3% | 296 s at 170 °C | 149 | —— | —— |  |
| transesterification | B2^[7a]^ | 2% | 11 s at 300 °C | 115 | 250 °C, 10 MPa for 5 min | —— |  |
| Schiff base | MB-PACM^[22]^ | 2.5% | 15.3 s at 220 °C | 103 | 180 °C, 15 MPa for 20 min | —— |  |
| acetal | EGTE0.7^[23]^ | 2% | 20 s at 180 °C | 88.9 | 120 °C, 10 MPa for 20 min | 160 °C with a 2.16 kg load |  |
| Guanidine Metathesis | 6^[24]^ | 1% | 46、29 and 43 s at 185 °C | 130 | —— | —— |  |
| Hindered amide | TMHA2^[25]^ | 5% | —— | 97.85 | 140 °C, 10 MPa for 10 min | 140 °C with a 60-rpm shear rate |  |
| pyrazole-urea | PPzU 7c^[26]^ | 5% | —— | 102.41 | 130 °C, 10 MPa for 30 min | —— |  |
| boronic ester | PU1.1^[27]^ | 1% | —— | 130 | 150 °C, 1 MPa for 30 min | —— |  |
| carbamate | POU6b^[3c]^ | 10% | —— | 107.84 | 120 °C, 10 MPa for 30 min | —— |  |
| C=C metathesis | PCC-B8^[10]^ | —— | 106.2 s at 180 °C | 66.9 | 170 °C, 30 MPa for 10 min | —— |  |
| disulfide bond | polymer 3^[28]^ | 5% | 79 ± 4 s at 150 °C | 78 | 150 °C, 5-10 MPa for 30 min | —— |  |
| vinylogous urethane | VU-N_80_O_20_^[29]^ | 1% | —— | 99.3 | —— | —— |  |

**Supplementary References**

[20] Z. Yu, S. Ma, Z. Tang, Y. Liu, X. Xu, Q. Li, K. Zhang, B. Wang, S. Wang, J. Zhu, *Green Chem.* **2021**, *23*, 6566-6575.

[21] M. Capelot, M. M. Unterlass, F. i. Tournilhac, L. Leibler, *ACS Macro Lett.* **2012**, *1*, 789-792.

[22] S. Wang, S. Ma, Q. Li, X. Xu, B. Wang, W. Yuan, S. Zhou, S. You, J. Zhu, *Green Chem.* **2019**, *21*, 1484-1497.

[23] Q. Li, S. Ma, P. Li, B. Wang, Z. Yu, H. Feng, Y. Liu, J. Zhu, *Macromolecules* **2021**, *54*, 8423-8434.

[24] A. J. Melchor Banales, M. B. Larsen, *ACS Macro Lett.* **2020**, *9*, 937-943.

[25] J. Zhang, Y. Zhou, S. Ma, F. Zhou, *Green Chem.* **2024**, *26*, 3368-3377.

[26] W. Liu, Z. Yang, Z. Qiao, L. Zhang, N. Zhao, S. Luo, J. Xu, *Nat. Commun.* **2019**, *10*, 4753-4761.

[27] X. Zhang, S. Wang, Z. Jiang, Y. Li, X. Jing, *J. Am. Chem. Soc.* **2020**, *142*, 21852-21860.

[28] D. J. Fortman, R. L. Snyder, D. T. Sheppard, W. R. Dichtel, *ACS Macro Lett.* **2018**, *7*, 1226-1231.

[29] S. p. Engelen, N. D. Dolinski, C. Chen, E. Ghimire, C. A. Lindberg, A. E. Crolais, N. Nitta, J. M. Winne, S. J. Rowan, F. E. Du Prez, *Angew. Chem. Int. Ed.* **2024**, *63*, e202318412.
